# High Level of Serum Tissue Kallikrein Is Associated with Favorable Outcome in Acute Ischemic Stroke Patients

**DOI:** 10.1155/2019/5289715

**Published:** 2019-06-02

**Authors:** Fei Wu, Yifeng Ling, Lumeng Yang, Xin Cheng, Qiang Dong, Wenjie Cao

**Affiliations:** ^1^Department of Neurology, Huashan Hospital, Fudan University, Shanghai, China; ^2^State Key Laboratory of Medical Neurobiology, Fudan University, Shanghai, China

## Abstract

**Background/Objectives:**

We sought to assess the association between a serum tissue kallikrein (TK) level and a 90-day outcome in acute ischemic stroke (AIS) patients who received acute reperfusion therapy.

**Methods:**

Consecutive AIS patients within 6 hours after stroke onset between December 2015 and August 2017 were prospectively recruited. Blood samples were collected before acute reperfusion therapy for serum TK measurement. Outcome was modified Rankin scale (mRS) score at 90 days after stroke onset. Binary logistic regression was performed to analyze the association between the baseline TK level and the clinical outcome.

**Results:**

Between December 2015 and August 2017, 75 patients (age range from 33 to 91 years, 72.0% male) were recruited in this study. Higher baseline TK was independently associated with a favorable functional outcome (mRS 0-2) (odds ratio 1.01, 95% confidence interval (CI) 1.00-1.02, *p* = 0.047) and low mortality rate (odds ratio 0.98, 95% CI 0.96-1.00, *p* = 0.049) at 90 days. Increased TK level was associated with 90 d mRS (0-2) with area under the curve of 0.719 (95% CI 0.596-0.842; *p* = 0.002).

**Conclusions:**

Serum TK can be a promising predictor of clinical outcome in AIS patients who received acute reperfusion therapy.

## 1. Introduction

Tissue kallikrein (TK), an important component of the kallikrein-kinin system, belongs to a subgroup of serine proteinases and processes low molecular weight kininogen to release kinin peptide, which in turn activates bradykinin B1 and B2 receptors and triggers a host of biological effects, including regulation of blood pressure, smooth muscle contraction and relaxation, vascular cell growth, vascular permeability, inflammatory cascades, electrolyte balance, and pain induction [1, 2]. Numerous experimental studies have been done to explore the role of TK and demonstrated that TK can inhibit inflammation [3, 4], suppress the proliferation of vascular smooth muscle cells [5, 6], exert neuroprotective effects in oxygen-glucose-deprived cells as well as in the ischemic brain [7, 8], and enhance angiogenesis [9].

The value of TK in clinical practice has been evaluated in several studies. In a multicenter case-control study conducted in China, elevated TK level was negatively associated with incident and recurrence of stroke [10] and negatively associated with the severity of coronary artery disease (CAD) [11]. Exogenous TK treatment significantly reduced the incidence of in-stent restenosis after middle cerebral artery stenting [12, 13]. However, the association between TK and outcome of acute ischemic stroke (AIS) remains unclear. We aimed to explore the relationship between the serum TK level and the 90-day functional outcome in AIS patients after reperfusion therapy.

## 2. Materials and Methods

### 2.1. Patients

Between December 2015 and August 2017, we prospectively recruited acute ischemic stroke (AIS) patients presenting to our institution within 6 hours of the onset. All of them received intravenous tissue plasminogen activator (IV-tPA) and/or endovascular therapy (EVT). Clinical data including baseline National Institutes of Health Stroke Scale (NIHSS), age, sex, and risk factors was obtained. Large artery occlusion (LVO) was defined as occlusion in the basilar artery, intracranial internal carotid artery, and M1-M2 segment of the middle cerebral artery (MCA). The institutional ethics committee approved the study, and written informed consent was obtained from patients or a legal representative before enrollment.

### 2.2. Serum TK Measurement

Blood samples were collected before acute reperfusion therapy. All blood samples were centrifuged immediately and stored at -80°C until tested. We used a quantitative sandwich enzyme-linked immunosorbent assay with the DuoSet Human Kallikrein 1 ELISA kit (catalog: DY2337-05; R&D System, Inc., USA).

### 2.3. Outcome Measurement

The primary outcome was disability at day 90 (3-month visit), as assessed by means of the modified Rankin scale (mRS) scores, dichotomized as favorable outcome (score of 0-2) or unfavorable outcome (score of 3-6) [14]. The secondary outcome was mortality within 90 days of stroke onset and hemorrhagic transformation (HT) during the hospitalization. HT was defined according to the European Cooperative Acute Stroke Study on CT as an area of increased attenuation within an area of low attenuation in a typical vascular distribution [15]. On MRI, HT was identified by the presence of blood-product signal characteristics on T1, T2, and gradient-echo sequences [16]. Among patients who initiated EVT, modified Thrombolysis in Cerebral Infarction (mTICI) was used as the reperfusion grading scale [17]. Good reperfusion was defined as mTICI 2b/3 [18].

### 2.4. Statistical Analysis

Statistical analyses were performed using SPSS, version 22 (SPSS Inc., Chicago, IL). *p* value less than 0.05 was considered to indicate statistical significance. Differences in patients' characteristics between groups were tested by the chi-square test for categorical and Mann-Whitney or *t* test for continuous values. Binary logistic regression (including variables with *p* < 0.15) was used to assess the association of variables with favorable outcome. Receiver operating characteristic (ROC) analysis was performed to determine the optimal threshold of TK in predicting 90-day mRS 0-2 or mortality.

## 3. Results

From December 2015 to August 2017, a total of 163 patients within 6 hours of symptom onset received IV-tPA and/or EVT in our institution. Seventy-five patients (age range from 33 to 91 years, 72.0% male) who signed the informed consent and finished 90-day follow-up were included in the final analysis. Fifty-three patients received IV-tPA only, 13 were bridged EVT, 6 were given EVT directly, and 3 undertook digital subtraction angiography only. Sixty-four percent (48/75) of patients achieved a favorable functional outcome (mRS 0-2) and 13.3% (10/75) of patients died within 90 days after stroke onset. LVO was identified in 48% (36/75) of patients.

### 3.1. Baseline TK Level and Outcomes in All Patients

Patients' characteristics in total and stratified by outcomes are listed in Table 1. Age (*p* < 0.001), history of atrial fibrillation (*p* = 0.021), low baseline NIHSS score (*p* < 0.001), and high baseline TK level (*p* = 0.002) were significantly associated with a good functional outcome (90 d mRS 0-2). Diabetes (*p* = 0.019), previous history of stroke (*p* = 0.014), high baseline NIHSS (*p* = 0.007), and low baseline TK level (*p* = 0.012) were significantly associated with 90 d mortality. No association was found between the baseline TK level and HT (*p* = 0.592).

In the binary logistic regression analysis of factors with *p* value less than 0.15, high baseline TK level was still significantly associated with a favorable functional outcome (odds ratio 1.01, 95% confidence interval (CI) 1.00-1.02, *p* = 0.047) and low rate of mortality at 90-day follow-up (odds ratio 0.98, 95% CI 0.96-1.00, *p* = 0.049) (Table 2).

In receiver operating characteristic analysis, increased baseline TK level was associated with 90 d mRS (0-2) with area under the curve (AUC) of 0.719 (95% CI 0.596-0.842; *p* = 0.002) and alive outcome with AUC of 0.748 (95% CI 0.567-0.928; *p* = 0.012). The optimal threshold of the baseline TK level determined by Youden's index was 24.92 pg/ml for functional outcome and 23.26 pg/ml for alive outcome. The threshold of baseline TK ≥ 25 pg/ml predicted a good functional outcome with a sensitivity of 66.7%, specificity of 77.8%, positive predictive value of 84.2%, and negative predictive value of 56.8%.

### 3.2. Baseline TK Level and Outcome in Subgroup Analysis

**Figure 1:**
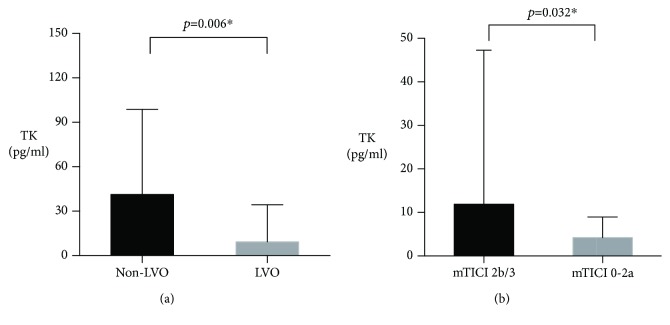
Comparison of TK levels in different subgroups. Abbreviations: TK: tissue kallikrein; LV: large vessel occlusion; mTICI: modified Thrombolysis in Cerebral Infarction. ^∗^*p* < 0.05. Data are presented as median with interquartile range.

**Figure 2:**
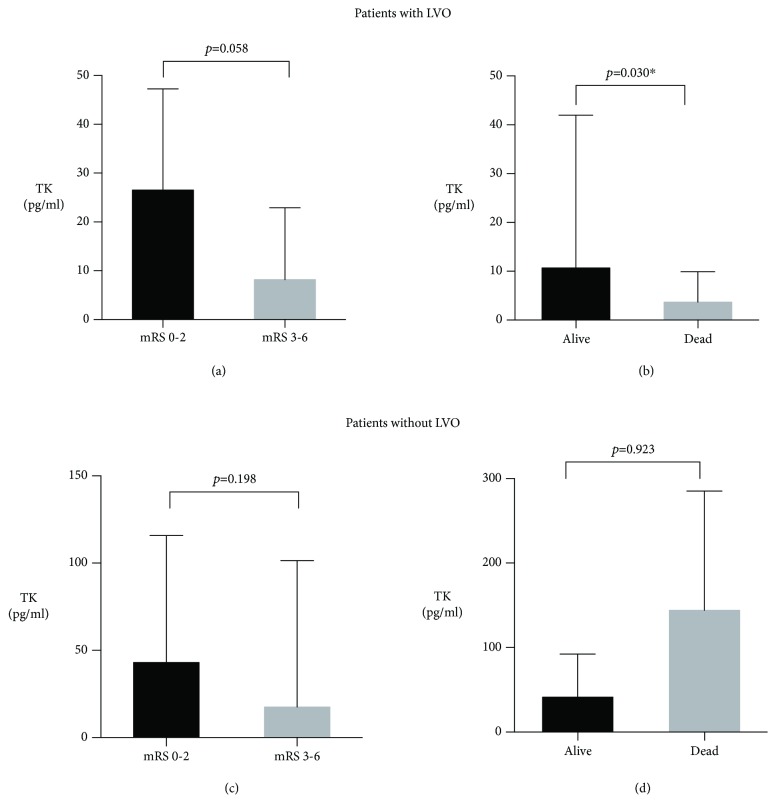
The association between the TK level and the outcomes in patients with or without LVO. Abbreviations: TK: tissue kallikrein; LVO: large vessel occlusion; mRS: modified Rankin Scale. ^∗^*p* < 0.05. Data are presented as median with interquartile range.

LVO was identified in 48% (36/75) of patients. The concentration of baseline TK in patients with LVO was significantly lower than patients without LVO (9.3 pg/ml vs. 41.3 pg/ml, *p* = 0.006) (Figure 1(a)). Baseline TK was significantly associated with mortality rate in patients with LVO (*p* = 0.030) while not in patients without LVO (*p* = 0.923). The relationship between baseline TK and favorable functional outcome was near to be significant in patients with LVO (*p* = 0.058) while not in patients without LVO (*p* = 0.198), seen in Figure 2.

Fifty-three patients received IV-tPA only. Subgroup analysis stratified by different kinds of acute treatment showed that high baseline TK level remained significantly associated with a favorable functional outcome (*p* = 0.019) and low mortality rate (*p* = 0.040) 90 days after ischemic stroke in patients that received IV-tPA only.

### 3.3. TK and Good Reperfusion

A total of 22 patients initiated EVT, and postoperative mTICI was assessed. Seven patients had intracranial carotid artery occlusion. Twelve patients had MCA occlusion, and three patients had basilar artery occlusion. Sixty-eight percent (15/22) of patients achieved good perfusion (mTICI 2b/3). Higher baseline TK level was significantly associated with good reperfusion (*p* = 0.032) (Figure 1(b)).

## 4. Discussion

The major finding of this study was that high level of TK before acute reperfusion therapy was significantly associated with a favorable functional outcome and low mortality rate at 90 days in AIS patients. The optimal threshold of TK for predicting a good functional outcome is 25 pg/ml. Lower level of baseline TK was seen in patients with LVO than those without. Higher baseline TK level was significantly associated with good reperfusion after EVT.

TK has been demonstrated to be associated with several diseases, especially cardiovascular disease and stroke. People with hypertension [19], heart failure [20], or coronary artery disease (CAD) [21] were found to have low levels of urinary human TK activity compared with controls. However, another study proposed that higher levels of TK in plasma were associated with the presence of CAD and were a predictor of mild coronary arteriosclerosis [11]. In stroke patients, both the plasma TK level and the urinary TK activity have a significant inverse relationship with first-ever stroke and stroke recurrence in the Chinese population [10, 22]. However, previous studies did not evaluate the predictive value of TK in AIS patients. Our study found that higher baseline TK level in serum is independently associated with a favorable 90 d outcome in AIS patients with IV-tPA and/or EVT.

It has been reported that TK was involved in the pathological course of cerebral ischemia/reperfusion [23, 24]. TK gene transfer is protected against cerebral infarction by promoting glial cell survival, migration, and inhibiting apoptosis [25]. Moreover, exogenous TK enhanced neurogenesis and angiogenesis in the subventricular zone and the periinfarction region and improved the neurological function after focal cortical infarction [3, 26]. Newly formed endothelial cells produce various vascular growth factors and neurotrophic factors such as brain-derived neurotrophic factor, vascular endothelial growth factor, and basic fibroblast growth factor. Consequently, these neurotrophic factors foster neural stem cell survival, which in turn improves neurological function [26]. Consistent with previous reports, we further demonstrated the positive correlation between endogenous TK and functional outcome in a human cohort.

Another study in rats has further evaluated whether TK improves neurological function after cerebral ischemia/reperfusion through its effects on the regional cerebral blood flow (rCBF) [27]. They found that the overexpression of TK gene elevated the rCBF significantly. The change was time-independent, and it was more notable at 72 h after treatment [27]. In our study, we adopted mTICI as the reperfusion grading scale and assessed the association between endogenous TK and mTICI 2b/3. We found that patients with higher level of baseline TK level would be more likely to achieve good reperfusion, which was in accordance with the previous study.

In acute LVO patients, we found a significantly lower level of TK than non-LVO patients. That phenomenon suggests it may be beneficial to increase the serum kallikrein in AIS patients with LVO. Human urinary kallidinogenase injection (HUK) is a glycoprotein extracted from men's urine approved by China's State Food and Drug Administration. A systematic review [28] including 24 trials (2433 patients) was conducted to assess the efficacy and safety of HUK in treating patients with AIS. The results suggested that HUK injection could reduce neurological impairment and improve long-term outcomes. However, only two trials reported death or dependence at the end of three-month follow-up, and none of the trials was conducted in AIS patients receiving acute reperfusion therapy with LVO. Another study reported forty-four cases with AIS between 6 and 72 h of onset that were randomly assigned into the kallikrein group (*n* = 24) and the control group (*n* = 20) [29]. They found that kallikrein improved neural function effectively and quickly after stroke, and promoting cerebral reorganization might be an important mechanism for kallikrein in the treatment of AIS; however, no difference was found in a 90 d outcome between groups. More strictly designed studies with large sample sizes are needed to evaluate whether treatment with TK could effectively and safely improve the functional outcome in AIS patients with LVO.

Limitations of our study were due to the small sample size and derived from a single-center experience. Results of our study could only be applied in AIS patients receiving IV-tPA and/or EVT. Those without IV-tPA and/or EVT were not recruited, which may impair the power of the results. In our study, there is a significant difference between TK and clinical outcomes. However, the statistic power is low because the odds ratio is near 1 and the *p* value is near 0.05, which may be due to the limited sample sizes. More studies were needed to validate our findings and explore the clinical application of TK in acute ischemic stroke patients, especially those with large artery occlusion.

## 5. Conclusions

Serum TK can be a promising predictor of clinical outcome in AIS patients.

## Figures and Tables

**Table 1:** Baseline clinical characteristics of patients stratified by outcomes.

Variables	mRS 0-2	mRS 3-6	*p* value	Alive	Dead	*p* value
	48	27		65	10	
Age (y)	63.3 ± 10.5	73.3 ± 9.5	<0.001	65.9 ± 11.3	73.4 ± 8.8	0.050
Male (%)	72.9 (35/48)	70.4 (19/27)	0.814	75.4 (49/65)	50.0 (5/10)	0.131
HBP (%)	72.9 (35/48)	74.1 (20/27)	0.913	69.2 (45/65)	100.0 (10/10)	0.054
DM (%)	20.8 (10/48)	37.0 (10/27)	0.128	21.5 (14/65)	60.0 (6/10)	0.019
Smoking (%)	45.8 (22/48)	40.7 (11/27)	0.670	46.2 (30/65)	30.0 (3/10)	0.497
AF (%)	16.7 (8/48)	40.7 (11/27)	0.021	21.5 (14/65)	50.0 (5/10)	0.110
Previous stroke	18.8 (9/48)	37.0 (10/27)	0.080	20.0 (13/65)	60.0 (6/10)	0.014
NIHSS	7 (3, 12)	14 (9, 20)	<0.001	8 (4, 12)	17 (10, 22)	0.007
OTT (min)	185 (122, 222)	150 (124, 192)	0.178	164 (123, 220)	164 (125, 198)	0.969
Baseline TK (pg/ml)	40.4 (9.2, 82.9)	8.4 (3.2, 23.3)	0.002	34.5 (7.7, 61.8)	3.7 (3.0, 13.3)	0.012
IV-tPA (%)	91.7 (44/48)	81.5 (22/27)	0.269	89.2 (58/65)	80.0 (8/10)	0.344
EVT (%)	18.8 (9/48)	37.0 (10/27)	0.080	26.2 (17/65)	20.0 (2/10)	1.000

Abbreviations: HBP: hypertension; DM: diabetes mellitus; AF: atrial fibrillation; NIHSS: National Institutes of Health Stroke Scale; OTT: onset-to-treatment time; TK: tissue kallikrein; IV-tPA: intravenous tissue plasminogen activator; EVT: endovascular treatment.

**Table 2:** Binary logistic regression analysis.

Predictors	OR	95% CI	*p* value
*Favorable functional outcome (90-day mRS 0-2)*			
Age	0.87	0.79-0.96	0.004
Baseline NIHSS	0.87	0.76-0.99	0.037
Baseline TK	1.01	1.00-1.02	0.047
AF	3.18	0.52-19.44	0.210
DM	0.33	0.08-1.42	0.136
Previous stroke	0.35	0.07-1.79	0.205
EVT	0.31	0.06-1.67	0.177

*90-day mortality*			
Age	1.16	0.95-1.40	0.137
Baseline NIHSS	1.05	0.94-1.17	0.371
Baseline TK	0.98	0.96-1.00	0.049
AF	3.44	0.36-32.77	0.283
HBP	1.465E+9		0.998
DM	15.78	0.95-261.1	0.054
Previous stroke	6.42	0.59-69.42	0.126

Abbreviations: OR: odds ratio; CI: confidence interval; NIHSS: National Institutes of Health Stroke Scale; TK: tissue kallikrein; AF: atrial fibrillation; DM: diabetes mellitus; EVT: endovascular therapy; HBP: hypertension.

## Data Availability

The clinical and imaging data used to support the findings of this study are available from the corresponding author upon request.
